# Ultra-High Sensitivity Zinc Oxide Nanocombs for On-Chip Room Temperature Carbon Monoxide Sensing

**DOI:** 10.3390/s150408919

**Published:** 2015-04-16

**Authors:** Xiaofang Pan, Xiaojin Zhao

**Affiliations:** 1 College of Information Engineering, Shenzhen University, Shenzhen 518060, China; E-Mail: eexpan@163.com; 2 College of Electronic Science and Technology, Shenzhen University, Shenzhen 518060, China; 3 Department of ECE, the Hong Kong University of Science and Technology, Clear Water Bay, Hong Kong, China

**Keywords:** room temperature, CO gas sensor, CMOS compatible, ultra-high sensitivity

## Abstract

In this paper, we report an on-chip gas sensor based on novel zinc oxide (ZnO) nanocombs for carbon monoxide (CO) sensing. With ZnO gas sensing nanocombs fully integrated on a single silicon chip, the concept of low cost complementary-metal-oxide-semiconductor (CMOS) microsensor capable of on-chip gas sensing and processing is enabled. Compared with all previous implementations, the proposed ZnO nanocombs feature much larger effective sensing area and exhibit ultra-high sensitivity even at the room temperature. Specifically, at room temperature, we demonstrate peak sensitivities as high as 7.22 and 8.93 for CO concentrations of 250 ppm and 500 ppm, respectively. As a result, by operating the proposed ZnO-nanocomb-based gas sensor at the room temperature, the widely adopted power consuming heating components are completely removed. This leads to not only great power saving, but also full compatibility between the gas sensor and the on-chip circuitry in term of acceptable operating temperature. In addition, the reported fast response/recovery time of ~200 s/~50 s (250 ppm CO) makes it well suited to real-life applications.

## 1. Introduction

The integration of gas sensing devices over a single complementary-metal-oxide-semiconductor (CMOS) integrated circuit (IC) chip enables the promising concept of on-chip gas sensing and processing [1]. It is known that an electronic olfactory system on a single chip provides significant advantages in terms of system miniaturization, manufacturing cost, sensing/processing speed, to name but a few [2,3]. Among all previously demonstrated gas sensor implementations [4,5,6,7,8], two major obstacles remain for the monolithic integration between gas sensor and CMOS IC chip: (1) manufacturing process incompatibility [4,5,6]; (2) high operation temperature and the needed high power consumption [7,8]. It is known that many gas sensors require high temperatures. Specifically, the widely-adopted micro-electro-mechanical systems (MEMS) process for fabricating gas sensor is typically not compatible with standard CMOS process [9,10]. In addition, the operation temperature of MEMS gas sensors (above 300 °C) is far beyond the maximum acceptable operating temperature of a wide range of standard CMOS IC chips [2]. More importantly, even the IC chips based on some CMOS processes can withstand this high operation temperature, the required additional on-chip/off-chip heating component still consumes high power, which significantly limits the sensors’ applications especially when they are adopted for mobile gas sensing systems powered by the batteries.

To alleviate the above obstacles and realize the so-called on-chip gas sensing and processing, a promising avenue is to directly pattern a layer of semiconductor metal oxide (SMO) material on top of the patterned electrodes of CMOS IC substrate [11]. Featuring full compatibility with standard CMOS process, SMO has enabled a wide range of gas sensing applications since it was first reported to exhibit conduct metric changes in reactive gas ambient [12]. Moreover, the feature size of SMO gas sensor layer is scaled down to nanometer level in order to maximize its effective sensing area (also known as “surface-to-volume ratio”), which largely determines the SMO gas sensors’ performance in terms of several crucial figures of merit, such as sensitivity, response/recovery time [13,14].

A number of SMO gas sensor implementations have been reported in the literature [8,9,10,11,15]. In [11], gas sensors based on networked ZnO nanowires were proposed to enhance both the sensitivity and the response. Although successful detection of low concentration NO_2_ is demonstrated, operating temperature as high as 225 °C is still necessary. In [15], operating at a temperature of 300 °C, S.K. Lim *et al.* reported a ZnO-nanorods-based gas sensor (prepared by ethyl benzene acid sodium salt) exhibiting a sensitivity of 50% for 100 ppm CO. The obvious drawback of the above approaches is that high temperature (200~300 °C) is inevitable to obtain acceptable sensitivity/signal to noise ratio (SNR). As a result, the associated power-hungry heating process is too prohibitive for the envisioned low power gas sensing microchip. Additionally, long-time operating at temperatures beyond 125 °C can dramatically downgrade the CMOS IC chips’ reliability (especially for the bulk and the epitaxial circuit) [2]. 

In this paper, novel ZnO nanocomb-based gas sensors are fabricated on a single silicon chip for on-chip carbon monoxide (CO) sensing. The proposed ZnO nanocombs feature room-temperature operation, and completely remove the widely exploited power-hungry heating components, leading to a great amount of power saving. Compared with previously reported SMO gas sensors, the proposed ZnO nanocomb exhibit a greatly enhanced sensitivity even at the room temperature. In addition, short response/recovery time is reported, which makes the high speed on-chip gas sensing/processing possible. The remainder of this paper is organized as follows: Section 2 and Section 3 present the principle and the fabrication process of the proposed ZnO nanocomb-based gas sensor. Experimental results are reported and discussed in Section 4. Finally, a conclusion is drawn in Section 5.

## 2. Sensing Mechanism

SMO can be used to sense a series of environmental parameters, including light, gas, pressure, humidity, to name but a few [16,17,18]. Regarding the gas sensing applications, a well-known property of SMO is that its surface can absorb oxygen molecules when exposed to an oxidizing gas environment, such as the air with 21% oxygen gas. Specifically, during the sensing process, the oxygen surface vacancy behaves as an absorption site, and oxygen ions can be formed by combining the oxygen molecules and the electrons from the SMO’s conduction band [19]. Depending on the operating temperature *T*, different chemical combinations can occur. Here the combination occurring at the room temperature is expressed as follows [20]:

(1) $$O_{2}+e^{-}\to {O_{2}}^{-}$$

With the conduction band’s electrons decreased, the original depletion region in vacuum is expanded and the sensing material’s overall resistance is increased until a new equilibrium is achieved. For the real-life applications, the newly established equilibrium, representing the response to air, is typically exploited as the SMO gas sensors’ idle status. As shown in Equation (2), when exposed to the incoming target gas (e.g., CO in this paper), this equilibrium will be broken and the incoming CO will react with the generated oxygen ions to release the combined electrons back to the conduction band, leading to significantly narrowed depletion region and decreased resistance. Thus, by measuring the SMO’s overall resistance in real time, the target CO gas’ concentration can be quantitatively detected.

(2) $${O_{2}}^{-}+2CO\to 2CO_{2}+e^{-}$$

**Figure 1:**
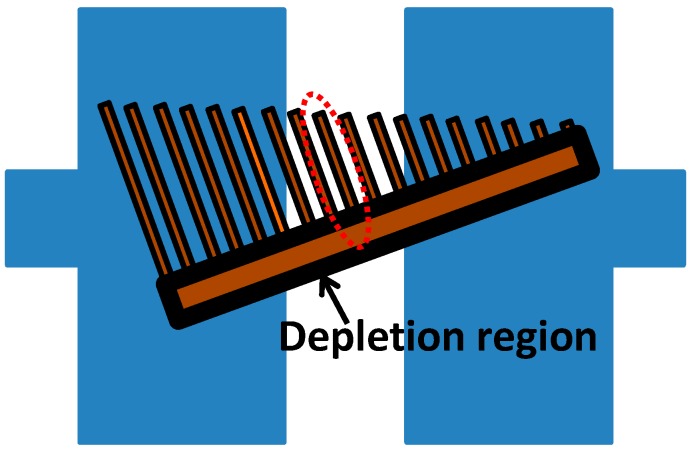
Proposed zinc oxide (ZnO) nanocomb based carbon monoxide (CO) gas sensor with two electrodes.

In this paper, we propose a novel ZnO nanocomb structure for sensing CO gas. As shown in Figure 1, the proposed nanocomb-based CO gas sensor is grown on top of a standard silicon substrate. In order to measure the ZnO nanocomb’s real-time overall resistance, dual-layer electrodes (Ti/Au) are deposited and patterned. Compared with the traditional widely-adopted nanowire structure, this novel nanocomb structure features multiple conducting channels and significantly elevated effective sensing area, leading to much larger surface to volume ratio. In addition, we would like to point out that the feature size of the adopted nanocomb is comparable to its surface depletion region’s width variation. This optimized design enables not only the significant improvement of the CO gas sensing performance (e.g., the sensitivity and the response time), but also the room temperature gas sensing without the need of any additional power-hungry heating component.

**Figure 2:**
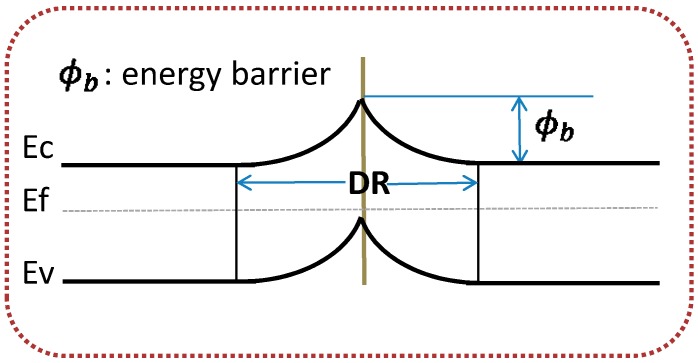
Energy diagram of the proposed ZnO nanocomb based gas sensor.

Figure 2 illustrates the proposed ZnO nanocomb structure’s energy diagram. It is observed that an energy barrier is formed at each intersection point of the nanocomb, which is quite different from the traditional nanowire structure [21]. According to [22], we can have the intersection point’s resistance exponentially influenced by this energy barrier:

(3) $$R=R_{0}\exp\left(\frac{\varphi_{b}}{KT}\right)=\frac{1}{gq\mu N_{d}}\exp\left(\frac{\varphi_{b}}{KT}\right)$$

where *φ_b_* is the energy barrier height, *g* is a semiconductor-geometry-determined constant, *μ* is the electrons’ mobility, *N_d_* is the donors’ density, *K* is Boltzman’s constant and *T* is the operating temperature, respectively. As a result, at different intersections, the final carrier distribution and the resistance are well balanced between the depletion region and the formed energy barriers.

Furthermore, according to Equation (3), it is worthy to mention that any incoming-gas-caused change of the energy barrier height can exert an exponential variation on the nanocomb’s overall resistance, which results in much faster response compared to its nanowire counterpart.

## 3. Device Fabrication

**Figure 3:**
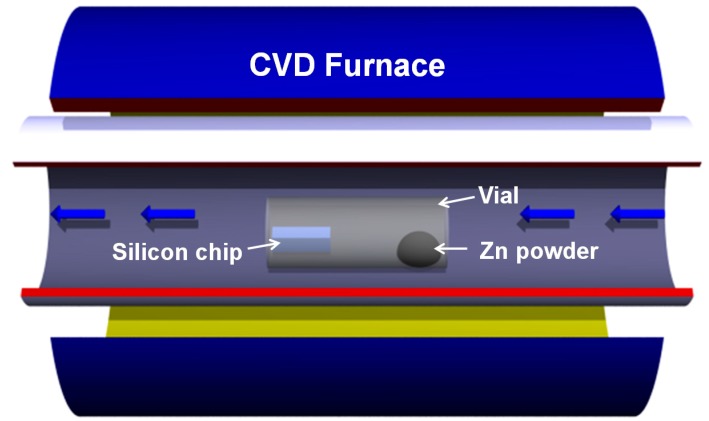
Illustration of our proposed ZnO nanocomb fabrication process.

In order to validate the proposed SMO gas sensing structure, we synthesize the ZnO nanocombs with vapor-liquid-solid (VLS)-mechanism-based chemical vapor deposition (CVD), which are composed of two major steps: (1) Au nanoparticle catalyst saturated with vaporized Zn and (2) ZnO precipitation with the reactive oxygen gas [23]. It is found that the ratio between vaporized Zn and reactive oxygen gas is crucial to the nucleation and final morphology of ZnO nanocombs. As shown in Figure 3, a vial is placed in the middle of the quartz tube to create a quasi-hermetic environment. As a result, the proposed nanocomb morphology can be produced with the well-controlled Zn/O_2_ ratio. 

Detailed fabrication steps are summarized as follows:

1. An Au layer with 2 nm thickness is evaporated on top of a clean silicon substrate as the catalyst of the following CVD process.
2. The evaporated Au catalyst is annealed at 700 °C for 30 min to form a layer of Au nanopaticles.
3. Half a gram of Zn powder (99.9%, from Sigma-Aldrich Inc.) is placed at the bottom of the vial as the source material; while the previously prepared silicon substrate with Au catalyst is mounted at the bunghole of the vial (Figure 3).
4. The furnace temperature is then increased to 700 °C. As a result, the Zn powder is vaporized.
5. A mixture of oxygen gas and argon gas (with a ratio of 1:49) is continuously blown into the furnace for 20 min under a pressure of 2.4 × 10^−^^3^ Torr.
6. The furnace temperature is cooled-down to room temperature. The silicon substrate appears white due to the densely deposited ZnO nanocombs.
7. The silicon substrate is immerged into isopropyl alcohol (IPA) solution to exfoliate the deposited ZnO nanocombs. In order to facilitate the exfoliation, it is always accompanied by light sonication.
8. The IPA solution with diluted ZnO nanocombs is dripped on a prefabricated SiO_2_/p-Si substrate (1 μm SiO_2_) with patterned Ti/Au electrode array on top, which is composed of a layer of Ti (10 nm) and a layer of Au (90 nm). Lift-off technique is utilized to pattern this dual layer electrode array with a 2 μm gap formed between each electrode pair.
9. Finally, the SiO_2_/p-Si substrate is annealed at 300 °C for 30 min to form ohmic contacts between the nanocomb’ two ends and the metal electrodes.

Moreover, we would like to point out that the post-processing of the electrodes will be required for CMOS integration, and for most advanced CMOS processes, copper (Cu) is adopted as the interconnection metal. In this paper, we focus on the fabrication/characterization of the proposed ZnO nanocombs, and more importantly, the exploration of its sensing performance’s upper-limit. Therefore, as in most previous literatures, Au is first used as the catalyst for growing the ZnO nanocombs, which enables a fair comparison with the previously reported SMO-based implementations. Efforts on replacing Au by copper as the catalyst are still ongoing.

## 4. Experimental Results and Discussions

**Figure 4:**
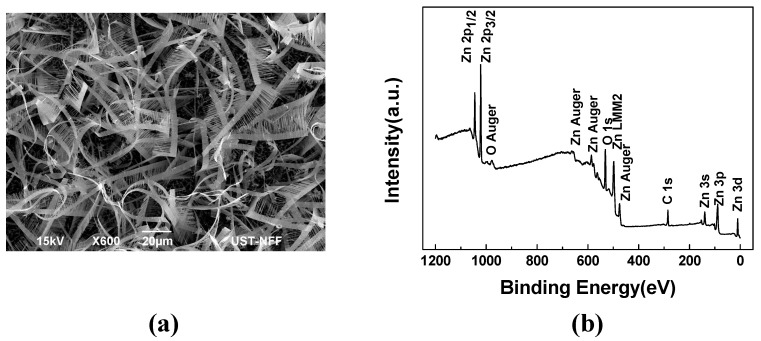
(**a**) The SEM picture of the fabricated ZnO nanocombs; (**b**) XPS characterization of the fabricated ZnO nanocombs.

In this section, we first examine the morphology of the fabricated ZnO nanocomb by using scanning electron microscopy (SEM). Figure 4a presents the SEM photograph of the fabricated ZnO nanocombs on the silicon substrate before their exfoliation. The length and the width of the nanocomb were measured to be ~58.7 μm and ~4.35 μm, respectively. After the fabricated method described in Section 3, nanocombs are dissolved in IPA solution by ultrasonic exfoliation, then the nanocombs are deposited onto the substrate with pre-fabricated electrodes having 2 μm gap. Figure 4b presents the XPS characteristics of the fabricated ZnO nanocombs.

**Figure 5:**
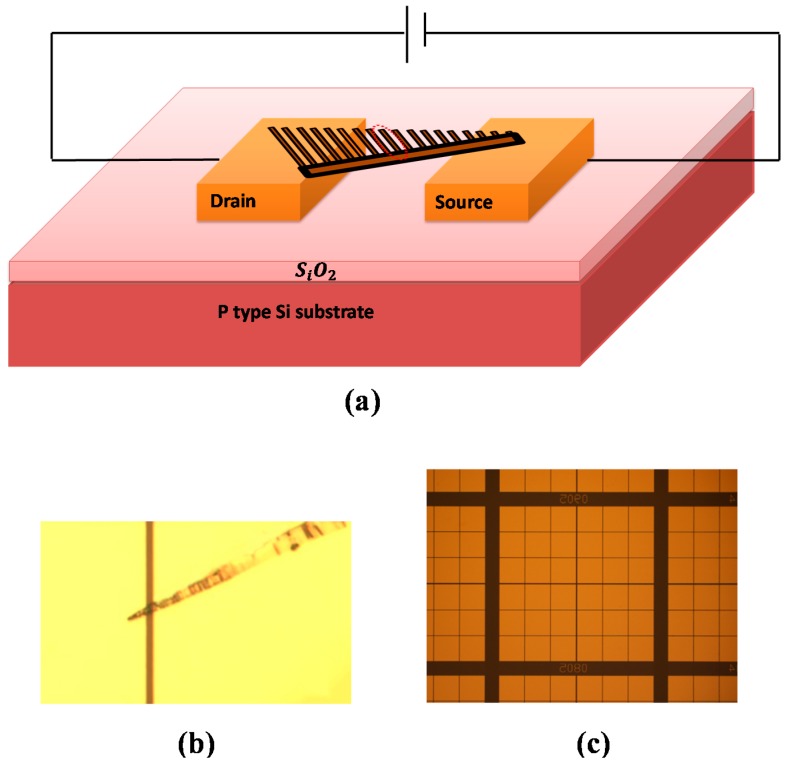
(**a**) Illustration of the ZnO nanocomb based quasi-field-effect-transistor (FET) device; (**b**) photo of the ZnO nanocomb device; (**c**) photo of the patterned electrodes.

**Figure 6:**
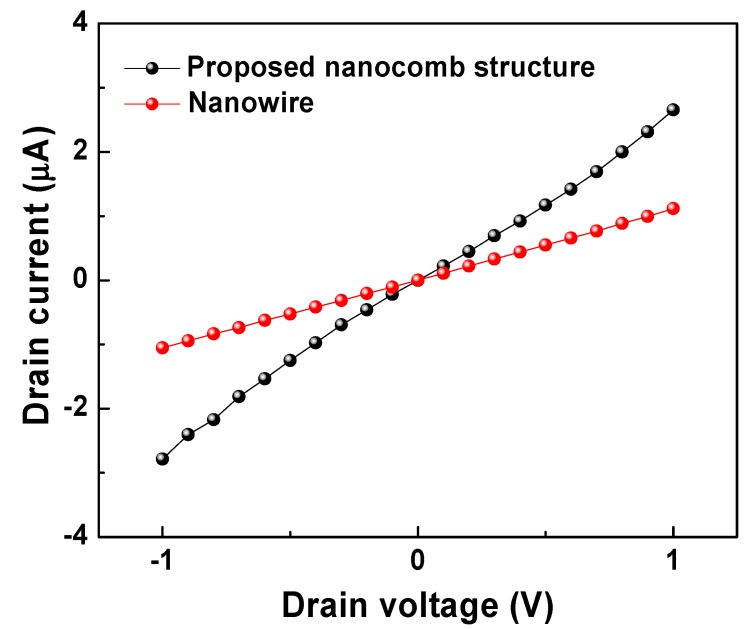
FET drain current comparison between the nanocomb and the nanowire.

In addition, our fabricated ZnO nanocombs are characterized through two approaches: (1) quasi-field-effect-transistor (FET) electrical properties with a semiconductor parameter analyzer from HP Corp. (HP4145B) and (2) temporal gas sensing performance. To study the electrical properties of the ZnO nanocomb, a quasi-FET device (Figure 5) composed of metal electrodes and ZnO nanocomb is prepared with the process flow described in Section 3, where the electrodes are composed of Ti and Au. With the electrodes patterned as Figure 5b,c, the gap between the adjacent electrodes is determined to be 2 μm. In addition, the electrode dimension is 100 μm × 100 μm, where the thickness of bottom layer (Ti) is 10 nm, and the thickness of top layer (Au) is 90 nm. Due to the work function mismatch between Au (5.31 eV) and ZnO (3.37 eV), a schottky contact is formed between the nanostructure and the metal electrode. As mentioned in the proposed fabrication process flow, the device is finally annealed at 300 °C for 30 mins to avoid the diode behavior of the schottky contact. Successively, the drain current was measured by sweeping the drain voltage from –1 V to 1 V with both the gate and the source grounded. In order to quantitatively reveal the proposed nanocomb’s advantages over the traditional nanowire, we fabricated and measured both nanocomb and its nanowire counterpart. As presented in Figure 6, for nanowire structure, the drain current exhibits a linear relationship with the drain voltage, representing a superior ohmic contact with the high temperature annealing process. In contrast, the nanocomb device shows higher conductivity compared with the nanowire device, which is mainly attributed to the nanocomb’s large surface to volume ratio. Moreover, we made altogether five devices, and the measured average resistance of the sensors is 0.33 MΩ, with a calculated sensor to sensor standard deviation (SD) of 0.053 MΩ.

Moreover, the fabricated ZnO nanocomb gas sensors’ temporal sensing performance is characterized by measuring three important figures of merit: sensitivity, response time and recovery time. Our sensor was measured with CO in the air. The air was pre-dried with its humidity lower than 10% RH. The current was measured with Keithley 2410. Regarding the conditioning stages of the sensor, we usually apply stable voltage until the current becomes stable, and this process typically takes 30 min. In addition, there were altogether 5 sensors tested and they were repeatedly tested.

**Figure 7:**
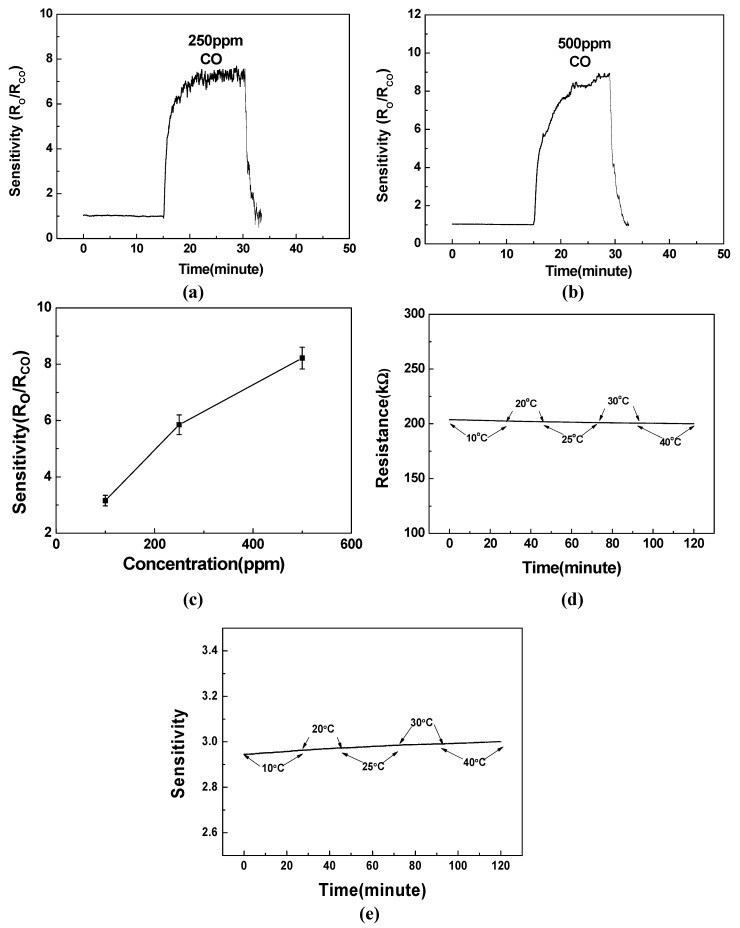
(**a**) Temporal CO sensing measurement results at the room temperature for 250 ppm CO; (**b**) temporal CO sensing measurement results at the room temperature for 500 ppm CO; (**c**) measured mean peak sensitivities with different CO concentrations; (**d**) measured temperature dependence of the fabricated gas sensor’s resistance; (**e**) measured temperature dependence of the fabricated gas sensor’s sensitivity.

As illustrated in Figure 7a,b, at the room temperature (*i.e.*, 25 °C with a variation of ±0.25 °C over the experimental period), the measured resistance for the idle status (*i.e.*, without target gas CO) is defined as *R_o_*. When exposed to CO gas with concentrations of 250 ppm and 500 ppm, the measured resistance *R_CO_* started to decrease and the calculated sensitivity started to increase. Here the gas sensor’s sensitivity is defined as the ratio between the measured nanocomb’s resistance without and with the target CO gas. As a result, the measured peak sensitivities are 7.22 and 8.93 for 250 ppm CO and 500 ppm CO, respectively. And the response time, defined as the time interval of the measured current increasing from 10% to 90% of the difference between the peak current and the base current, was measured to be 200 s and 400 s for CO gas with concentrations of 250 ppm and 500 ppm, respectively. Successively, by removing the injected CO gas, the measured current started to recover rapidly and the recovery time, defined as the time interval of the measured current decreasing from 90% to 10% of the difference between the peak current and the base current, was calculated to be 50 s and 55 s for CO gas with concentrations of 250 ppm and 500 ppm, respectively. Figure 7c shows the measured mean peak sensitivities with different CO concentrations. Furthermore, the temperature dependence of the fabricated sensor’s resistance is presented in the Figure 7d. As a result, we do not observe any significant dependence with the temperature ranging from 10 °C to 40 °C. According to the sensitivity definition (*i.e.*, R_o_/R_CO_), the temperature dependence of the sensitivity is shown in Figure 7e. Consequently, we still do not observe any significant temperature dependence. In order to disclose the advantages of our demonstrated CO gas sensor, we compare this work against the previously reported CO sensors in Table 1 [24,25,26,27,28,29]. It is observed that the proposed ZnO nanocomb-based gas sensor boasts recovery time of 50 s for 250 ppm CO and 55 s for 500 ppm CO, which is, to the best of our knowledge, the shortest recovery time ever reported. In addition, provided with the target gas having same concentration as [26] (*i.e.*, 500 ppm), the proposed sensor improves the response time by 200 s. However, it does not perform as well compared to [24,28,29]. This is partially attributed to the high peak sensitivities exhibited by our fabricated sensors, which needs extra response time to achieve this large resistance variation. As the sensitivity, our fabricated ZnO nanocomb takes great advantages for 250 ppm CO and 500 ppm CO when compared with [24,26]. For [25,27,28,29] with different measured CO concentrations (*i.e.*, 100 ppm, 1660 ppm, 260 ppm and 300 ppm), we would like to point out that the direct comparison of the sensitivity is quite challenging since this parameter is closely related to the concentration of the target gas. And the adopted different gas sensing materials/structures exhibit various sensitivity *vs.* concentration relationships, which prohibits the fair comparison even with linearly normalized target gas concentration. Nevertheless, we can still make the following observations with respect to [27,28,29], the sensitivities of reported Zinc ferrite particles and SnO_2_ thin film are limited to 2.22 and 1.5 respectively for CO gas with concentrations as high as 1660 ppm and 260 ppm. In [29], only a sensitivity of 5.5 is reported for the Co-doped ZnO nanorods exposed to 300 ppm CO gas. In this paper, our proposed implementation exhibits a sensitivity as high as 7.22 for the target CO gas with a lower concentration of 250 ppm. Finally, it is worthy to emphasize that all of our experimental results were acquired at the room temperature (*i.e.*, 25 °C), which can be greatly enhanced with the same operating temperature as applied in [24,25,26,27,28,29] (*i.e.*, 200~350 °C). More importantly, none of the previously reported implementations are capable of operating at the room temperature, which makes it necessary to include an additional heating component. This not only results in high power consumption overhead, but also brings a series of thermal reliability issues to the CMOS transistors after the real integration with CMOS IC chips.

**Table 1:** Gas sensing performance comparison with previous implementations.

Ref.	Materials	CO Concentration (ppm)	Operating Temperature (°C)	Peak Sensitivity	Response Time (s)	Recovery Time (s)	Additional Heating Component
[24]	Cu-doped ZnO thin film	250	300	4	50	100	Needed
[25]	Mesoporous In_2_O_3_ nanofiber	100	300	5.3	600	600	Needed
[26]	ZnO	500	200	2.8	600	120~180	Needed
[27]	Zinc ferrite particles	1660	350	2.22	Not reported	Not reported	Needed
[28]	SnO_2_ thin film sensor	260	350	1.5	28	94	Needed
[29]	Co-doped ZnO Nanorods	300	350	5.5	~60	~60	Needed
This work	ZnO nanocomb	250	25(RT)	7.22	200	50	No need
		500		8.93	400	55	

## 5. Conclusions

We have fabricated and characterized a ZnO-nanocomb-based gas sensor with ultra-low power consumption. The ultra-low overall power consumption is achieved by completely eliminating the need of additional heating components in the traditional implementations. Moreover, by operating our fabricated gas sensor at the room temperature, the proposed implementation exhibits superior gas sensing characteristics, including a high peak sensitivity of 8.93 (500 ppm CO gas), a short response time of 400 s and a short recovery time of 55 s. Meanwhile, the reported fabrication recipe is fully compatible with the standard CMOS process, which makes it well suited to the mass production of low cost smart gas sensing systems. Additionally, we are currently working on directly depositing and patterning a layer of functional SMO material (e.g., ZnO nanocomb) on top of the standard CMOS silicon chip’s passivated electrodes, with standard CVD and lift-off process. As mentioned above, for most advanced CMOS processes, copper (Cu) is adopted as the interconnection metal. Therefore, the primary issue is to explore the feasibility of using copper as the catalyst for growing our proposed ZnO nanocombs, and the efforts are still ongoing. Another big issue is the possible performance drift after long-term usage. In order to address this issue, we are developing dedicated calibration algorithms as well, which are all hardware-friendly and can be realized by the on-chip CMOS digital circuitry.
